# Emerging virus coinfections of the CNS: HTLV I/II as a common denominator

**DOI:** 10.1186/1471-2334-14-S2-P94

**Published:** 2014-05-23

**Authors:** George Panos, Maria Sargianou, Dionysios Watson, Nikolaos Dimisianos, Paraskevi Chra, Marina Andreou, Christina Sklavou, John Ellul

**Affiliations:** 1 Patras University General Hospital, Patras, Greece; 2 Athens General Hospital “Korgialenio-Benakio”, Greece

## Introduction

HTLV I is a retrovirus that can result in decreased effectiveness of immune response and an increased incidence of coinfections. HTLV I CNS coinfections with rare clinical manifestations that may relate to impaired cellular immunity are presented.

## Materials and methods

Case1: a 15-year-old patient presented with difficulty in standing up and walking on her own (following a fall), with visual disturbances, and refractory epileptic seizures; travel history included trips to the Caribbean (Isla Margarita).

Case2: a 24-year-old patient presented with difficulty in standing up and walking without support, with sudden hearing loss, peripheral paresthesia, and generalized weakness, after having experienced grand mal seizures several days before; travel history included trips to the Caribbean (her fiancé was captain of a cruise ship), the Amazon rainforest and Antarctica.

## Results

Case1: brain MRI imaging revealed localized, non-Gadolinium enhancing lesions in the right thalamus and occipital lobe and cortex edema. EBV DNA and HTLV I/II RNA were detected in the CSF and EBV VCA IgG antibodies in the serum. Following administration of supportive treatment and gancyclovir IV for EBV encephalitis, the patient was discharged with residual mobility impairment.

Case2: brain MRI imaging revealed several medium sized, non-Gadolinium enhancing lesions in the white matter of both hemispheres (especially in the temporal lobe). India ink examination and latex agglutination for Cryptococcus spp. were positive in the CSF. Cryptococcus gattii was consequently isolated from CSF cultures. HTLV I/II antibodies and HTLV I/II RNA were both detected in the serum only. Following a 4-month hospitalization, the patient was discharged with clinical improvement, but with relative speech and mobility impairment.

## Conclusions

Neurologic manifestations can be encountered in HTLV I/II monoinfected and coinfected patients, regardless of HAM/TSP. In CNS coinfections, the definite diagnosis for HAM/TSP cannot be established by current diagnostic criteria, reflecting the need for further criteria to also include coinfections. Quantitation of HTLV I/II proviral load and investigation of factors related to cellular immunity might augment diagnosis and prognosis.

